# Supplementary material for: Comparative efficacy and safety of antiresorptive and anabolic therapies for male osteoporosis: an updated Bayesian network meta-analysis
Source: Front Endocrinol (Lausanne). 2026 Apr 1;17:1714141. doi: 10.3389/fendo.2026.1714141 (PMC13079047; doi:10.3389/fendo.2026.1714141)

**Appendix.**

**Supplementary data**

**Search Strategy;**

**PUBMED (3117)**

((((((((((((((((((((((((((((((((((Alendronate[MeSH Terms]) OR (Aminohydroxybutane Bisphosphonate[Title/Abstract])) OR (4-Amino-1-Hydroxybutylidene 1,1-Biphosphonate[Title/Abstract])) OR (Fosamax[Title/Abstract])) OR (Alendronate Sodium[Title/Abstract])) OR (Alendronate Monosodium Salt, Trihydrate[Title/Abstract])) OR (Risedronic Acid[MeSH Terms])) OR (Bisphosphonate Risedronate Sodium[Title/Abstract])) OR (Risedronate Sodium, Bisphosphonate[Title/Abstract])) OR (Sodium, Bisphosphonate Risedronate[Title/Abstract])) OR (Risedronate Sodium[Title/Abstract])) OR (Actonel[Title/Abstract])) OR (Risedronic Acid, Monosodium Salt[Title/Abstract])) OR (Risedronate[Title/Abstract])) OR (1-Hydroxy-2-(3-pyridyl)ethylidene diphosphonate[Title/Abstract])) OR (Atelvia[Title/Abstract])) OR (2-(3-pyridinyl)-1-hydroxyethylidene-bisphosphonate[Title/Abstract])) OR (Zoledronic Acid[MeSH Terms])) OR (2-(Imidazol-1-yl)-1-hydroxyethylidene-1,1-bisphosphonic acid[Title/Abstract])) OR (Zoledronic Acid Anhydrous[Title/Abstract])) OR (Zometa[Title/Abstract])) OR (Zoledronate[Title/Abstract])) OR (Denosumab[MeSH Terms])) OR (AMG 162[Title/Abstract])) OR (Xgeva[Title/Abstract])) OR (Prolia[Title/Abstract])) OR (Teriparatide[MeSH Terms])) OR (hPTH (1-34[Title/Abstract]))) OR (Human Parathyroid Hormone (1-34[Title/Abstract]))) OR (Parathar[Title/Abstract])) OR (Teriparatide Acetate[Title/Abstract])) OR (Abaloparatide[MeSH Terms])) OR (BA058[Title/Abstract])) OR (tymlos[Title/Abstract])) AND ((((Osteoporosis[MeSH Terms]) OR (Osteoporoses[Title/Abstract])) OR (Osteoporosis, Age-Related[Title/Abstract])) OR (Osteoporosis, Age Related[Title/Abstract])) OR (Age-Related Osteoporosis[Title/Abstract])) OR (Age-Related Osteoporoses[Title/Abstract])) OR (Age Related Osteoporosis[Title/Abstract])) OR (Osteoporoses, Age-Related[Title/Abstract])) OR (Bone Loss, Age-Related[Title/Abstract])) OR (Age-Related Bone Loss[Title/Abstract])) OR (Age-Related Bone Losses[Title/Abstract])) OR (Bone Loss, Age Related[Title/Abstract])) OR (Bone Losses, Age-Related[Title/Abstract])) OR (Osteoporosis, Senile[Title/Abstract])) OR (Osteoporoses, Senile[Title/Abstract])) OR (Senile Osteoporoses[Title/Abstract])) OR (Senile Osteoporosis[Title/Abstract])) OR (Osteoporosis, Involutional[Title/Abstract])) OR (Osteoporosis, Post-Traumatic[Title/Abstract])) OR (Osteoporosis, Post Traumatic[Title/Abstract])) OR (Post-Traumatic Osteoporoses[Title/Abstract])) OR (Post-Traumatic Osteoporosis[Title/Abstract])) AND (((Men[MeSH Terms]) OR (Male[MeSH Terms])) OR (Males[Title/Abstract]))))

**Cochrane Central Register of Controlled Trials (309)**

#1 MeSH descriptor: [Osteoporosis] explode all trees

#2 MeSH descriptor: [Men] explode all trees

#3 MeSH descriptor: [Male] explode all trees

#4 #2 or #3

#5 #1 and #4

#6 MeSH descriptor: [Alendronate] explode all trees

#7 MeSH descriptor: [Risedronic Acid] explode all trees

#8 MeSH descriptor: [Zoledronic Acid] explode all trees

#9 MeSH descriptor: [Denosumab] explode all trees

#10 MeSH descriptor: [Teriparatide] explode all trees

#11 abaloparatide

#12 #6 OR #7 OR #8 OR #9 OR #10 OR #11

#13 #5 AND #12

**Web of science (1800)**

1: (((((((((((((((((TS=(Osteoporosis)) OR TS=(Osteoporoses)) OR TS=(Osteoporosis, Age-Related)) OR TS=(Osteoporosis, Age Related)) OR TS=(Age-Related Osteoporosis)) OR TS=(Age-Related Osteoporoses)) OR TS=(Age Related Osteoporosis)) OR TS=(Osteoporoses, Age-Related)) OR TS=(Bone Loss, Age-Related)) OR TS=(Age-Related Bone Loss)) OR TS=(Age-Related Bone Losses)) OR TS=(Bone Loss, Age Related)) OR TS=(Bone Losses, Age-Related)) OR TS=(Osteoporosis, Senile)) OR TS=(Osteoporoses, Senile)) OR TS=(Senile Osteoporoses)) OR TS=(Senile Osteoporosis)) OR TS=(Osteoporosis, Involutional)

2: (TS=(Men)) OR TS=(Male)

3:(((((((((((((((((((((((((((((TS=(Diphosphonates)) OR TS=(Bisphosphonate)) OR TS=(Bisphosphonates)) OR TS=(Alendronate)) OR TS=(Aminohydroxybutane Bisphosphonate)) OR TS=(4-Amino-1-Hydroxybutylidene 1,1-Biphosphonate)) OR TS=(Alendronate Sodium)) OR TS=(Alendronate Monosodium Salt, Trihydrate)) OR TS=(Risedronic Acid)) OR TS=(Bisphosphonate Risedronate Sodium)) OR TS=(Risedronate Sodium, Bisphosphonate)) OR TS=(Sodium, Bisphosphonate Risedronate)) OR TS=(Risedronate Sodium)) OR TS=(Ibandronic Acid)) OR TS=(Ibandronate)) OR TS=((1-Hydroxy-3-(methylpentylamino)propylidene)bisphosphonate)) OR TS=(1-Hydroxy-3-(methylpentylamino)propylidenebisphosphonate)) OR TS=(abaloparatide )) OR TS=(Tymlos)) OR TS=(Denosumab)) OR TS=(Xgeva)) OR TS=(Prolia)) OR TS=(Teriparatide)) OR TS=(hPTH (1-34))) OR TS=(Human Parathyroid Hormone (1-34))) OR TS=(Parathar)) OR TS=(Teriparatide Acetate)) OR TS=(Zoledronic Acid)) OR TS=(2-(Imidazol-1-yl)-1-hydroxyethylidene-1,1-bisphosphonic acid)) OR TS=(Zoledronic Acid Anhydrous)

4: ((((TS=(randomized controlled trial)) OR TS=(randomized )) OR TS=(Clinical Trials, Randomized)) OR TS=(Trials, Randomized Clinical)) OR TS=(Controlled Clinical Trials, Randomized)

5: #4 AND #3 AND #2 AND #1

**Supplementary Table 1.** Model Fit Assessment: If the difference in DIC values between two models is within 5, it indicates that the data is consistent. DIC stands for Deviance Information Criterion.

|  | The Global inconsistency | ＞0.05 | DIC of Model of consistency | DIC of Model of inconsistency | The difference is  less than 5 |
| --- | --- | --- | --- | --- | --- |
| Lumbar spine BMD | 0.1273 | yes | 42.58394 | 39.76628 | yes |
| Femoral neck BMD | 0.1938 | yes | 35.19275 | 36.89012 | yes |
| Total hip BMD | 0.2546 | yes | 28.47653 | 30.94467 | yes |
| All adverse events | 0.3182 | yes | 47.10938 | 45.23761 | yes |
| Serious adverse events | 0.4067 | yes | 21.38462 | 24.11257 | yes |

**Supplementary Table 2.** Risk of bias (RoB1) judgments by domain for included trials

|  | **Random sequence generation** | **Allocation concealment** | **Blinding of participants and personnel** | **Blinding of outcome assessment** | **Incomplete outcome data** | **Selective reporting** | **Other bias** |
| --- | --- | --- | --- | --- | --- | --- | --- |
| Boonen 2009 | Low | Low | Unclear | Low | Unclear | Low | Low |
| Boonen 2011 | Low | Low | Unclear | Low | High | Low | Low |
| Boonen 2012 | Low | Low | Unclear | Low | Unclear | Low | Low |
| Czerwinski 2022 | Unclear | Low | Low | Unclear | Unclear | Low | Low |
| Gonnelli 2003 | Low | Low | Low | High | Low | Low | Low |
| Hwang 2010 | Low | Unclear | Unclear | Low | Low | Low | Low |
| Matsumoto 2022 | Low | Unclear | Low | Unclear | Low | Low | Low |
| Miller 2004 | Unclear | Low | High | Low | Low | Low | Unclear |
| Orwoll 2000 | Low | Low | Unclear | Unclear | Low | Low | Unclear |
| Orwoll 2003 | Low | Low | Unclear | Low | Low | Low | Unclear |
| Orwoll 2010 | Low | Unclear | Unclear | Low | Low | Low | Low |
| Orwoll 2012 | Low | Unclear | Unclear | Low | Low | Low | Low |
| Ringe 2004 | Low | Low | Unclear | Low | Low | Unclear | Low |
| Ringe 2009 | Low | Low | Unclear | Low | Low | High | Low |
| Susan 2025 | Low | Low | Low | Unclear | Unclear | Low | Low |
| Walker 2013 | Low | Low | Unclear | Unclear | Low | Low | Low |

**Supplementary Figure 1.** The [forest map](javascript:;) of all outcomes.

a)The results of [forest map](javascript:;) for Femoral neck BMD.


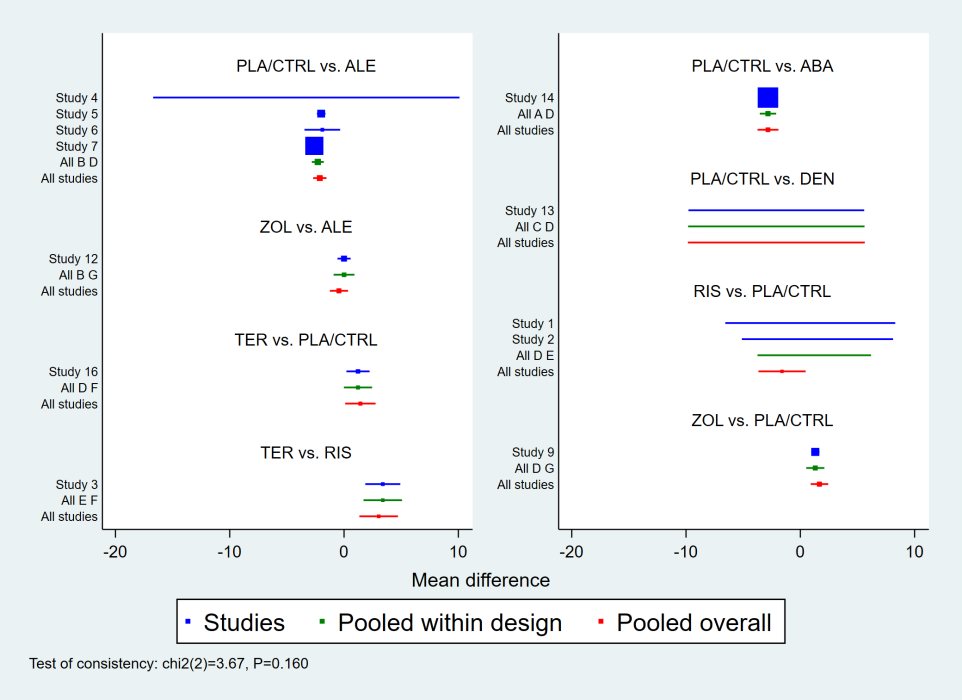


b) The results of [forest map](javascript:;) for Lumbar spine BMD.


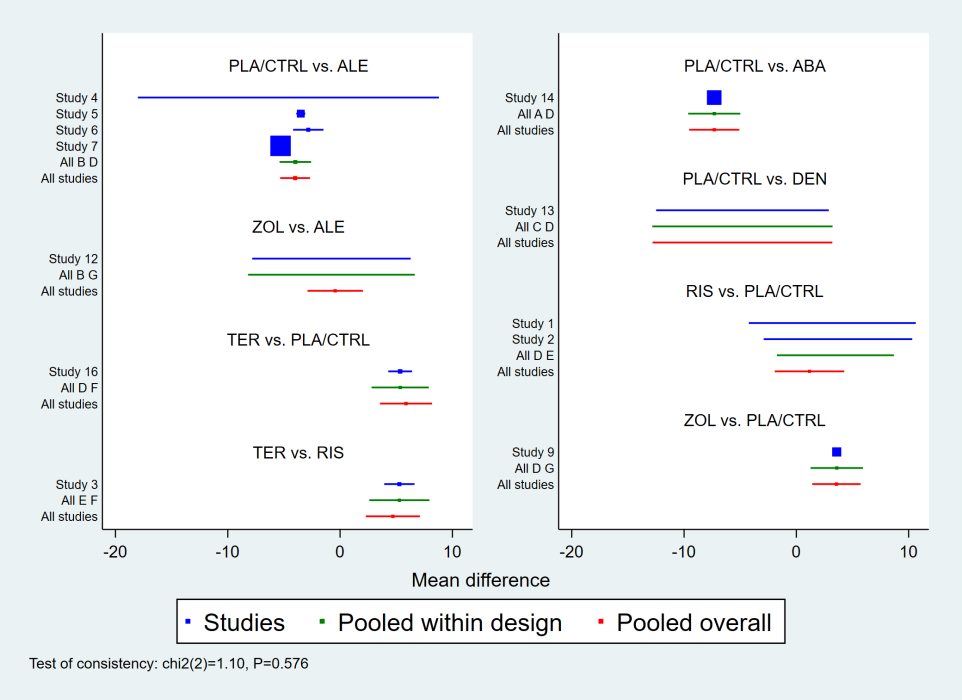


c) The results of [forest map](javascript:;) for Total hip BMD.


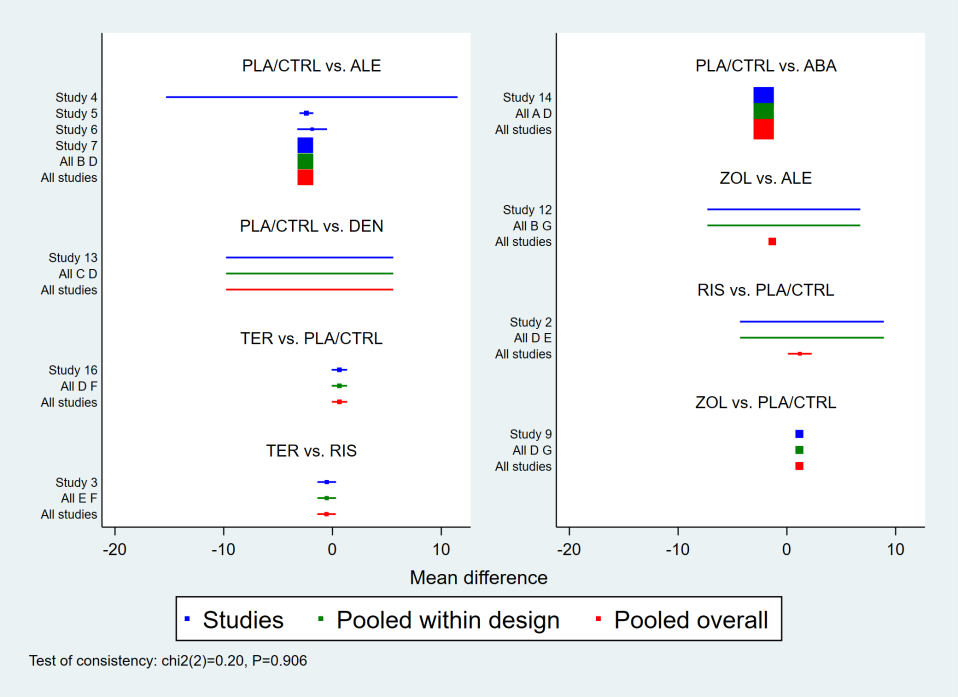


d) The results of [forest map](javascript:;) for All adverse events.


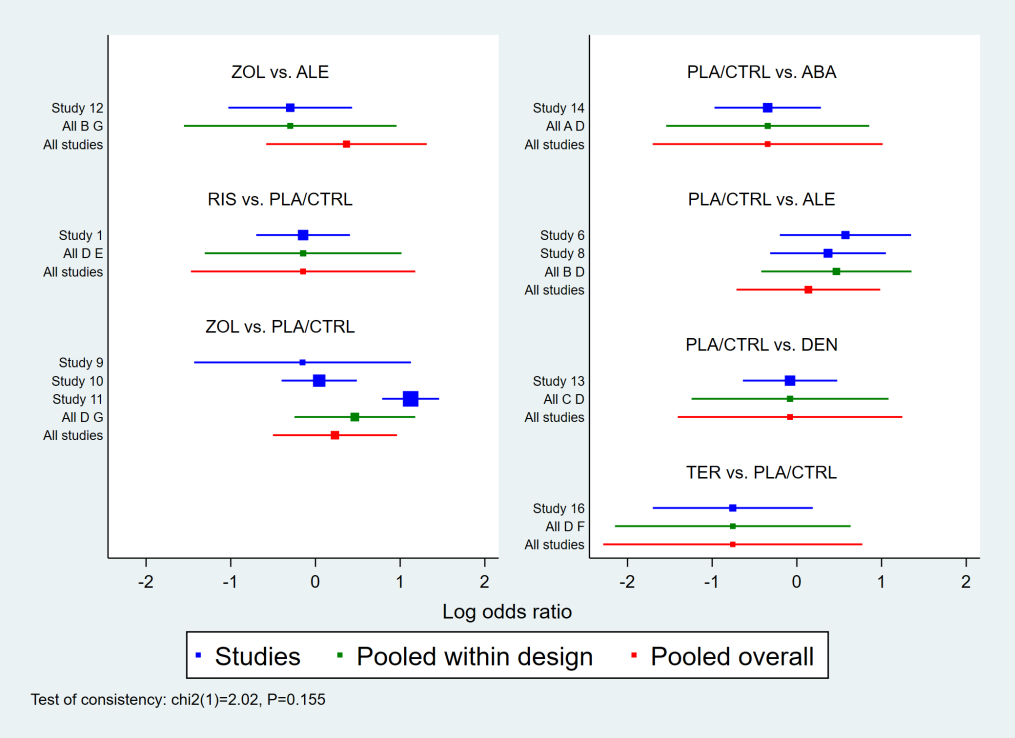


e) The results of [forest map](javascript:;) for Serious adverse events.


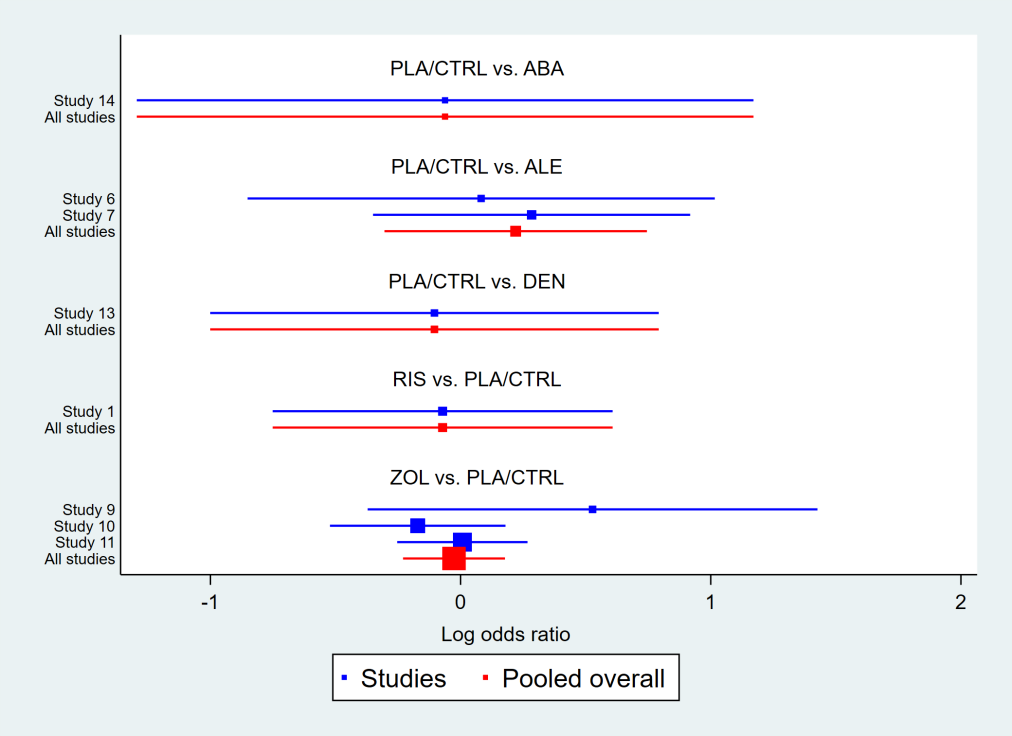


**Supplementary Figure 2.** The network plot of all outcomes.

1. Femoral neck BMD


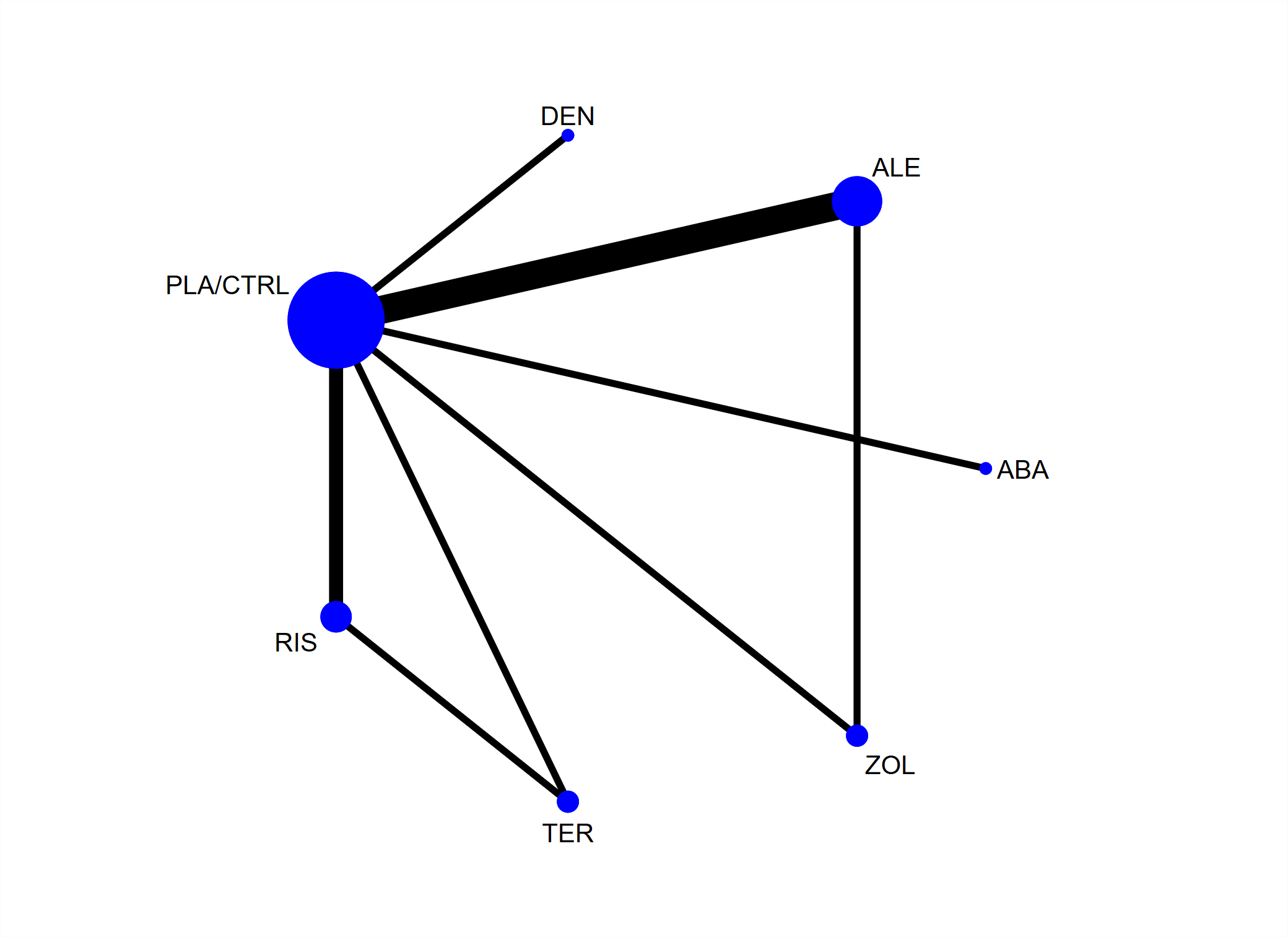


1. Lumbar spine BMD


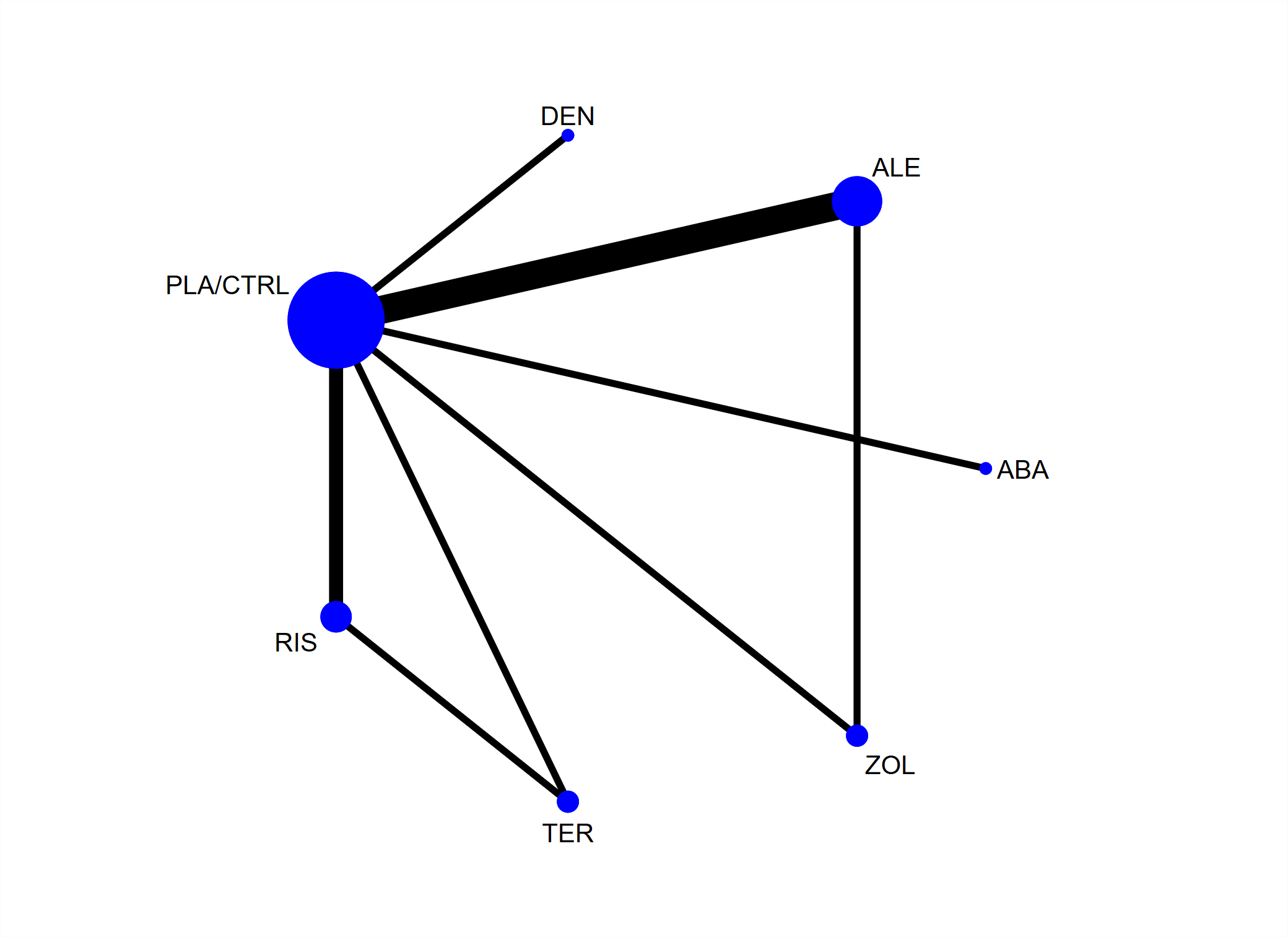


1. Total hip BMD


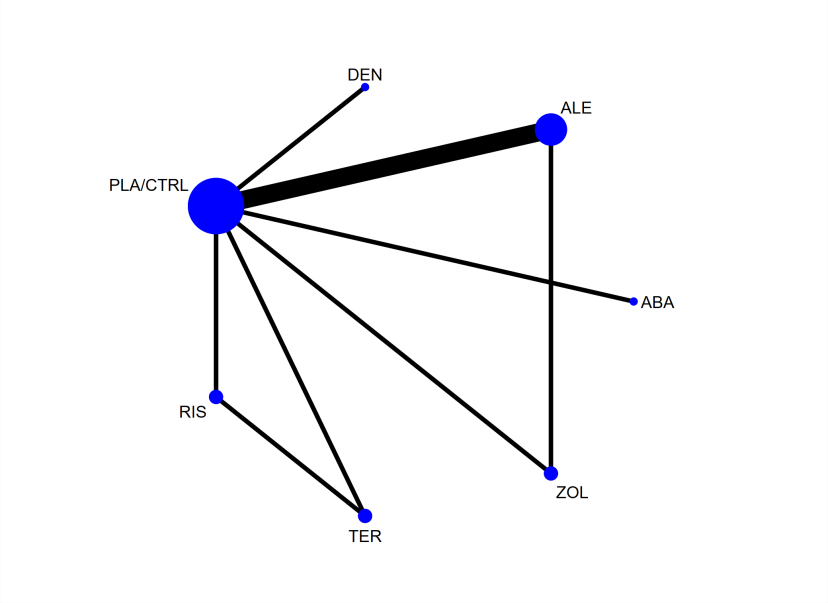


1. All adverse events


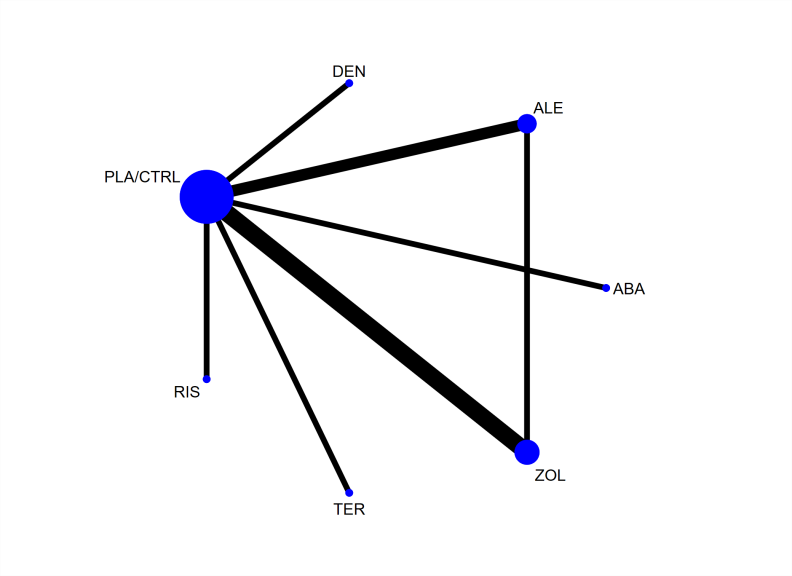


1. Serious adverse events


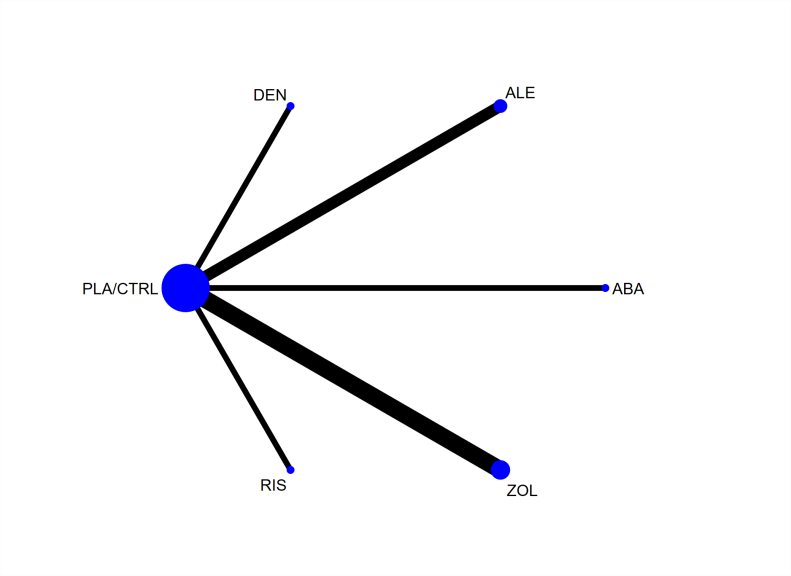


**Supplementary Figure 3.** The Funnel plot of all outcomes.

1. Femoral neck BMD


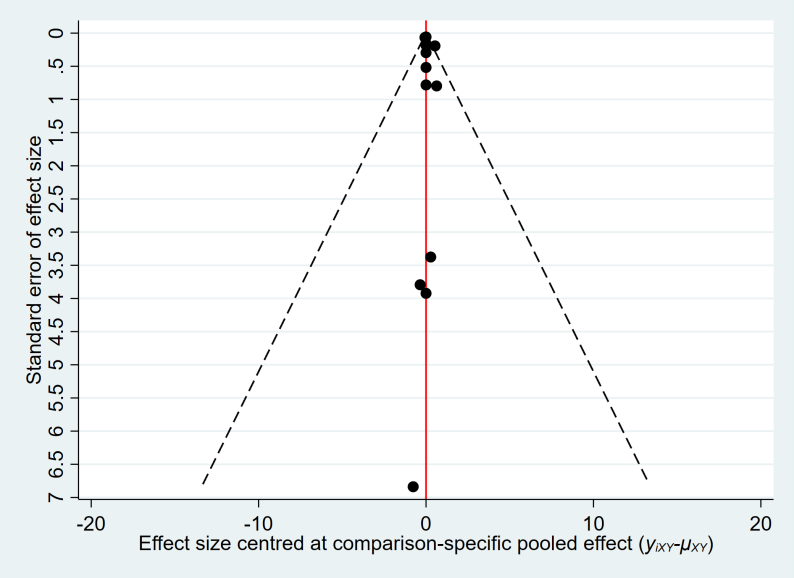


1. Lumbar spine BMD


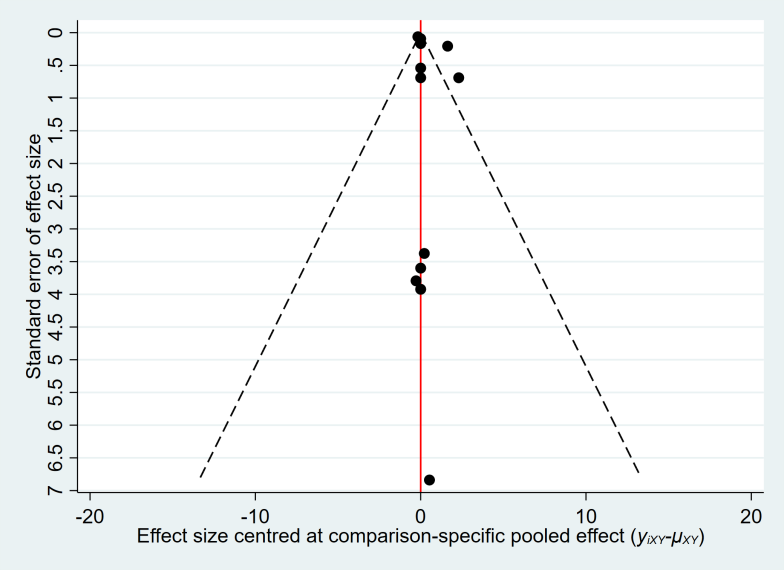


1. Total hip BMD


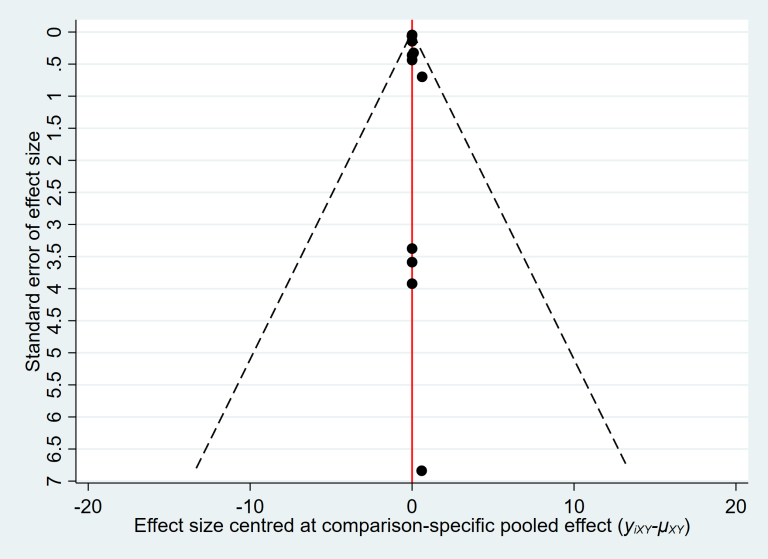


1. All adverse events


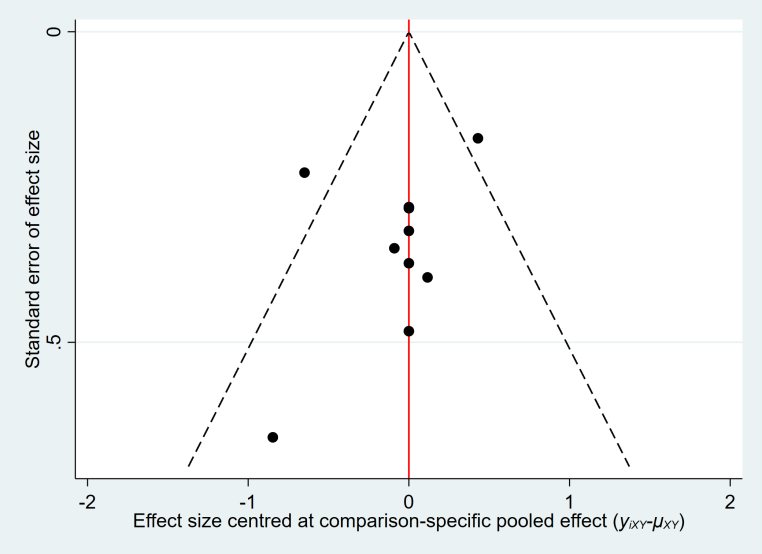


1. Serious adverse events


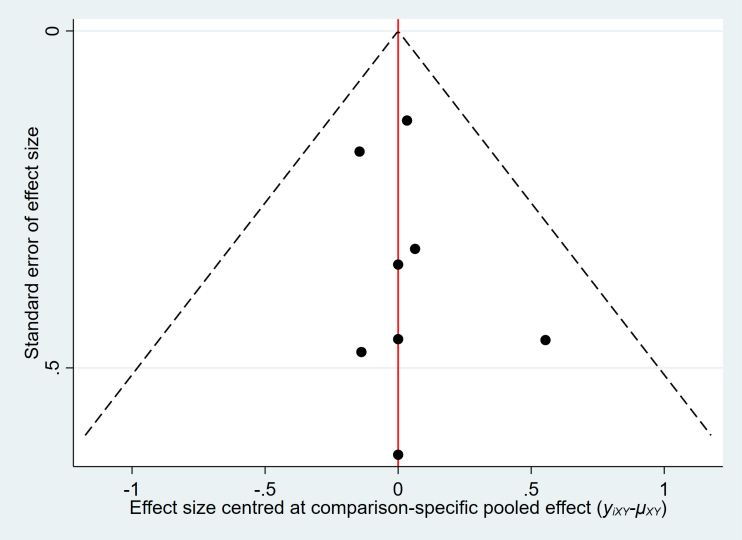

Supplement: Supplementary file 1 [file DataSheet1.docx]
